# A Chinese Mind-Body Exercise Improves Self-Control of Children with Autism: A Randomized Controlled Trial

**DOI:** 10.1371/journal.pone.0068184

**Published:** 2013-07-10

**Authors:** Agnes S. Chan, Sophia L. Sze, Nicolson Y. Siu, Eliza M. Lau, Mei-chun Cheung

**Affiliations:** 1 Neuropsychology Laboratory, Department of Psychology, The Chinese University of Hong Kong, Shatin, Hong Kong SAR, China; 2 Integrative Neuropsychological Rehabilitation Center, The Chinese University of Hong Kong, Shatin, Hong Kong SAR, China; 3 Henan Songshan Research Institute for Chanwuyi, Henan, China; 4 Institute of Textiles and Clothing, The Hong Kong Polytechnic University, Kowloon, Hong Kong SAR, China; University of Minnesota, United States of America

## Abstract

Self-control problems commonly manifest as temper outbursts and repetitive/rigid/impulsive behaviors, in children with autism spectrum disorders (ASD), which often contributes to learning difficulties and caregiver burden. The present study aims to compare the effect of a traditional Chinese *Chan*-based mind-body exercise, *Nei Yang Gong*, with that of the conventional Progressive Muscle Relaxation (PMR) technique in enhancing the self-control of children with ASD. Forty-six age- and IQ-matched ASD children were randomly assigned to receive group training in *Nei Yang Gong* (experimental group) or PMR (control group) twice per week for four weeks. The participants’ self-control was measured by three neuropsychological tests and parental rating on standardized questionnaires, and the underlying neural mechanism was assessed by the participants’ brain EEG activity during an inhibitory-control task before and after intervention. The results show that the experimental group demonstrated significantly greater improvement in self-control than the control group, which concurs with the parental reports of reduced autistic symptoms and increased control of temper and behaviors. In addition, the experimental group showed enhanced EEG activity in the anterior cingulate cortex, a region that mediates self-control, whereas the PMR group did not. The present findings support the potential application of Chinese *Chan*-based mind-body exercises as a form of neuropsychological rehabilitation for patients with self-control problems.

Chinese Clinical Trial Registry; Registration No.: ChiCTR-TRC-12002561; URL: www.chictr.org.

## Introduction

Executive dysfunction, which refers to difficulties in working memory, attention, planning, response inhibition, mental flexibility, and/or self-monitoring, is a typical cognitive deficit associated with autism spectrum disorders (ASD). Of these executive dysfunctions, response disinhibition and mental inflexibility are relatively more common in individuals with ASD [1]–[3]. It has also been suggested that executive dysfunctions accounts for typical autistic features, such as uncontrollable behavioral and emotional reactions, repetitive behaviors, strong need for sameness, restricted interests, and inappropriate social communication and interaction.

As there is not effective pharmacological intervention for enhancing the executive control of emotions and behaviors in individuals with ASD, the majority of the interventions are primarily behavioral [4]–[5]. Despite the reported effectiveness of some of the behavioral interventions in reducing behavioral or emotional disturbances, the intervention programs tend to be very intensive and time consuming [6]–[7]. For instance, although applied behavioral analysis was found to be effective in reducing various autistic symptoms, it requires intensive training for at least 20 hours per week for at least two years [7]. Furthermore, such positive outcomes are less prominent for children with limited mental ability or more severe autistic symptoms, who are not able to follow the rules and requirements of the training [8]. In view of the limitations of the conventional interventions, some researchers have been exploring various alternative methods, such as massage [9]–[11], acupuncture [12]–[13], music therapy [14]–[16], and diet/nutrition [17]–[19]. Although some preliminary data supports the positive effects of these novel interventions, the treatment efficacies remain largely inconclusive [20]. Our research team has been studying the effects of a traditional Chinese *Shaolin* medical concept (called *Chanyi*) in reducing some of the executive dysfunctions in individuals with ASD for the past years [21]–[24]. The present study further examines one component of this intervention approach, i.e., the mind-body exercise, as a possible intervention to improve the self-control of children with ASD.

According to the National Center for Complementary and Alternative Medicine (NCCAM) established by the US government, a mind-body intervention is defined as any intervention which “focuses on the interactions among the brain, mind, body and behavior, with the intent to use the mind to affect physical functioning and promote health”. The fundamental assumption of these interventions is that because the mind and the body are inter-connected, individuals have the internal ability to change their own thoughts and behaviors to enhance their mental and physical health. Mind-body interventions have long been used in China to improve both mental and physical health, and are now becoming increasingly popular in Western countries. Moreover, a range of scientific studies have examined the therapeutic effects of mind-body interventions on depression [25]–[26], anxiety [27], insomnia [28], chronic pain [29], and cardiovascular problems [30].

Although the existing studies have found encouraging empirical evidence for the treatment efficacy of mind-body interventions, the majority of the studies have examined adult or elderly populations. As a result, the effects and applicability of mind-body interventions on the pediatric population are relatively less understood. A few studies have explored the effects of relaxation training and mind-body interventions on the psychological and cognitive functions of children, and the results have so far been positive [31]–[38]. It should be noted that many of the mind-body techniques, such as meditation and *Tai Chi*, requires either a high degree of mental control or involves slow movements that might not be easily mastered by children with particular neurodevelopmental disorders. Nevertheless, the emerging positive outcomes of certain forms of mind-body training for normally developed children have provided insights into their potential effects on children with ASD.

The present study investigated the efficacy of the mind-body exercise, *Nei Yang Gong*, which is one component of a newly developed mind-body intervention. The intervention, which is based upon a Chinese *Chan* tradition named *Chanwuyi* (i.e., Zen, martial arts and healing) from the *Sanhuangzhai* monastery [39], is also known as the *Dejian* Mind-Body Intervention (DMBI), as named after the Grand Master of *Chanwuyi* – *Shi Dejian* (a *Shaolin* monk). Recent empirical studies on this intervention have shown that it has positive effects in improving physical health, mood and cognitive function in community-dwelling adults [40], children with autistic/Asperger’s disorder [22]–[24], and individuals with brain damage [41] and depression [25]–[26]. While the previous studies examined the effects of the holistic approach of *Chanyi*, which includes psychoeducation, mind-body exercises and diet modification, the present study examined the effect of a single component of the model, the mind-body exercises, as a possible intervention for children with ASD.

Somewhat like *Tai Chi*, *Nei Yang Gong* involves sets of slow movements that emphasize smooth, gentle, and calm movements. The basic principles and practices of *Nei Yang Gong* have been elaborated in two published books [39], [42] and on the website of a charity foundation (www.chanwuyi.org). The practice of *Nei Yang Gong* has two primary functions. First, it aims to foster self-awareness and mental self-control to help restore a calm and relaxed state. Second, it helps to reduce stress, increase flexibility of the limbs, and improve the circulation of *Qi* and blood. For example, the “shoulder relaxation” movement helps to relax the neck and the shoulders while improving self-control and self-awareness. It should be noted that *Nei Yang Gong* has been developed on the basis of the *Chan* medical model, which emphasizes the maintenance of a natural and relaxed attitude to achieve smooth circulation of *Qi* and blood. In this way, *Nei Yang Gong* differs from some of the other mind-body techniques, such as mindfulness and meditation, which require a high degree of conscious mental awareness and self-control. Given its required “natural” attitude, it is anticipated that *Nei Yang Gong* will be easily mastered by and feasibly applied to children with ASD. Our hundreds of clinical cases of children with neurodevelopmental disorders reveal that even children with moderate mental retardation are able to learn and benefit from *Nei Yang Gong.*

Our previous randomized controlled study revealed the possible neural mechanism underlying the therapeutic effect of *Nei Yang Gong* [43]. It was found that regular practice of *Nei Yang Gong* was able to foster a simultaneously relaxed and attentive brain state, as reflected in increased electroencephalographic (EEG) alpha asymmetry and intra- and inter-hemispheric EEG theta coherence indices. In contrast, this altered brain activity was not found among the participants of the comparison group, who practiced Progressive Muscle Relaxation (PMR). As relaxation and attentiveness are both significant factors for improving performance, *Nei Yang Gong* can be regarded as both a cognitive enhancement method and a relaxation exercise. Therefore, it is anticipated that the children who practice *Nei Yang Gong* (i.e., the experimental group) will show greater improvement in self-control (as measured by standardized neuropsychological tests) and greater reduction in related behavioral and social problems (as measured by parental reports on daily behaviors and social communication) than children who practice PMR (i.e., the control group). Derived from the work of Jacobson [44], PMR is a conventional and well-established behavioral treatment technique. It was selected for the comparison group because of the repeated empirical support for the effectiveness of PMR in alleviating anxiety, physiological arousal, and behavioral disturbances [45]–[47]. Furthermore, given the concreteness and simplicity of PMR, it can be applied to children with ASD at a younger age and with a lower functioning level. Scientific evidence supports the applicability and effectiveness of PMR in reducing aggression, increasing self-control and inducing relaxation in children as young as age 6 [48], [49] and in children with learning disabilities [50], [51]. Moreover, a few studies have reported that PMR alone or as a component of Cognitive Behavioral Therapy has positive effects in reducing anxiety and disruptive behavior of individuals with ASD [52]–[54].

The present study also compared the neural activity patterns of the two groups as measured by EEG assessment during an inhibitory control task (i.e., a Go/No-Go task) to explore the possible underlying neurological mechanism relating to improved self-control. The EEG test was adopted because it is non-invasive, practical, and easy to administer to children with ASD who have difficulty sitting still. The source of the scalp-EEG activity was localized using the standardized low-resolution brain electromagnetic tomography (sLORETA) analysis method [55] with the anterior cingulate cortex (ACC) as the region of interest. The ACC plays a significant role in inhibitory control [56]–[58] and reduced activity levels in the ACC have been found in children with ASD when performing an inhibitory task [59]–[61]. Accordingly, it is anticipated that if the children practicing *Nei Yang Gong* show improvement in self-control, then the source activity levels in the ACC during an inhibitory control task will be elevated, whereas this neurophysiological change will not be found in those practicing PMR.

## Materials and Methods

The protocol for this trial and supporting CONSORT checklist are available as supporting information; see Checklist S1 and Protocol S1.

### Study Design

**Figure 1:**
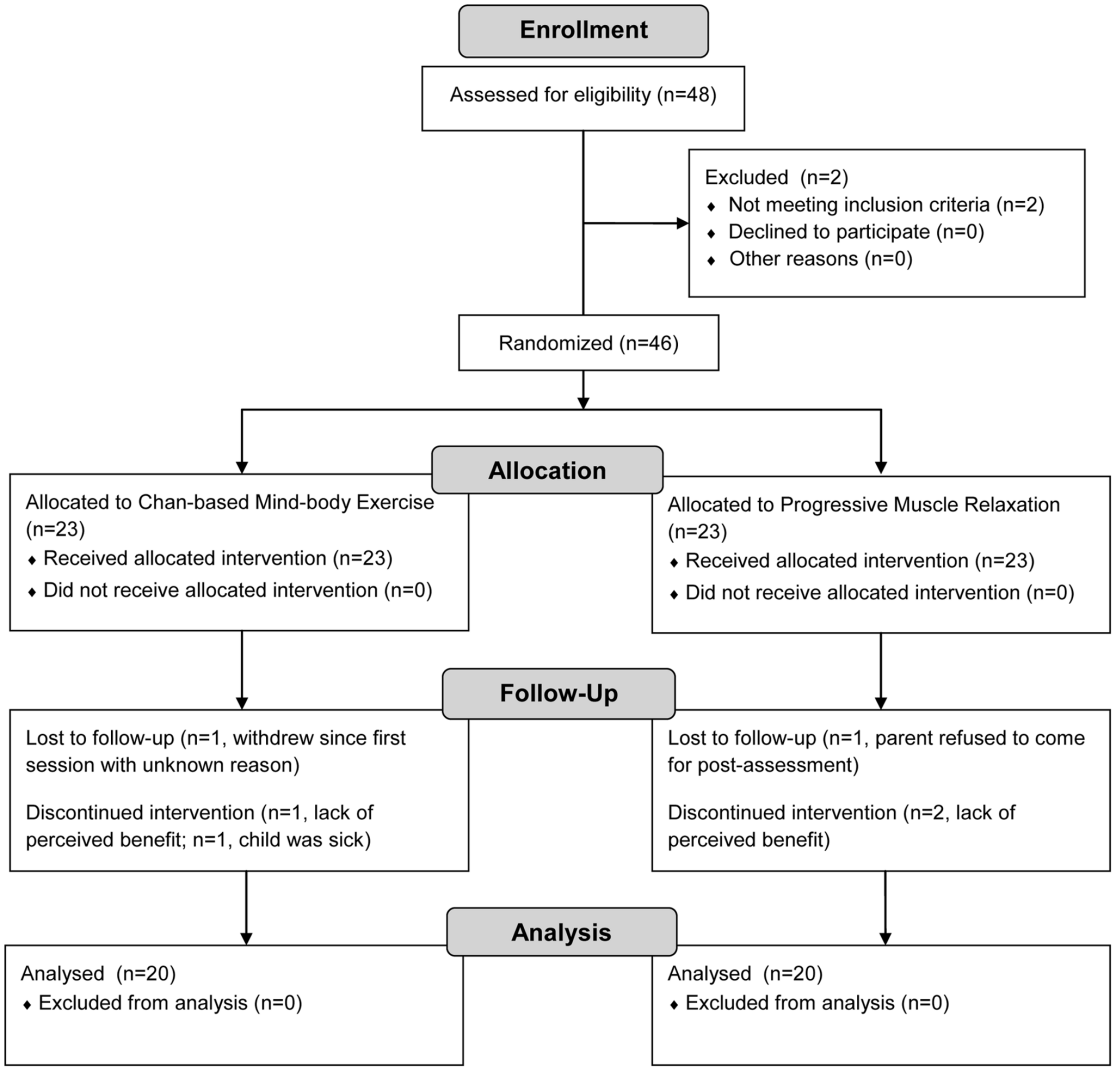
CONSORT flow diagram. The diagram shows the progress of the participants through the enrollment, allocation, follow-up, and data analysis phases of the trial.

The study was designed as a randomized controlled parallel trial. The recruited children with ASD were randomly and equally assigned into the experimental group (who received training on *Nei Yang Gong*), or the control group (who received training on Progressive Muscle Relaxation). Their ability to exercise self-control was measured before and after the one month of intervention. The progress of the participants throughout the trial is depicted in Figure 1.

### Ethics Statement

This study was conducted in accordance with the Helsinki Declaration of the World Medical Association Assembly. The research protocol was approved by the Human Subjects Ethics Sub-committee (HSESC) of the Hong Kong Polytechnic University (Ref. No.: HSEARS20120517001) and the Chinese Clinical Trial Registry (Registration No.: ChiCTR-TRC-12002561). Written consent was obtained from the parent of each participant prior to the commencement of the study.

### Participants

Forty-eight children with ASD aged between 6 and 17 voluntarily participated in the study with their parents’ written consent. Prior to the recruitment, the *a priori* sample size was estimated based on the effect size (0.7) of our previous research on this Chinese mind-body intervention for autism; with α = 0.05, power = 0.8, and the usual attrition rate in our previous studies of about 25%, the minimum required samples in each group was 24. The children were recruited from three primary schools and one secondary school in Hong Kong using an online advertisement and application platform, and the existing database at the Neuropsychology Laboratory of the Chinese University of Hong Kong. Except for two individuals, all participants received formal diagnosis of Autistic Disorder or Pervasive Developmental Disorders Not Otherwise Specified (PDD-NOS) by a clinical psychologist through a standard clinical interview with their parents based on the DSM-IV-TR criteria [62]. The clinical psychologist also assessed the severity of each child’s autistic symptoms in relation to social interaction, communication and repetitive/stereotypical behaviors using the Autism Diagnostic Interview-Revised (ADI-R) [63]. The interview included detailed questions about a child’s early development and current functioning, with higher scores indicating more severe autistic symptoms. Children with other neurodevelopmental, psychiatric or neurological comorbidities, or were on prescribed psychiatric medication, were excluded from the study.

The remaining 46 children were matched into pairs according to their age and level of intelligence. The individuals in each matched pair were then randomly assigned into the experimental or the control group with equal probability using the drawing lots method. The random assignment was performed by a research assistant who was blind to the experimental design. Before the end of the intervention, six children in total dropped out of the study, leaving 20 children each in the control and experimental groups. Table 1 presents the demographic and clinical characteristics of the two groups. The children in both groups were matched on age, *t*(38) = 1.01, *p* = 0.32, gender, *χ^2^*(1) = 1.11, *p* = 0.29, and severity of autistic symptoms as measured by the four ADI-R subscales, with *t* ranging from −0.80 to 0.97, and *p* ranging from 0.34 to 0.87. The two groups also demonstrated comparable levels of general intelligence, *t*(38) = 0.36, *p* = 0.72, which was assessed by the research assistant using the short form of the Chinese version of the Wechsler Intelligence Scale for Children-Fourth Edition (Hong Kong) (WISC-IV(HK)) [64] or the Stanford–Binet Intelligence Scale–Fourth Edition (SB-FE) [65] for non-verbal children and those who showed a floor effect in the WISC-IV(HK). Six children (i.e., 30% of the participants) in each group had limited intelligence with an IQ score below 70.

**Table 1:** Baseline Demographic and Clinical Characteristics of Participants in the Control and Experimental Groups.

Characteristics	Control Group	Experimental Group	*t/* *χ^2^*	*P* value
	(*n* = 20)	(*n* = 20)		
Age, years	12.42 (3.25)	11.28 (3.90)	1.01	0.32
Gender-Male (%)	85.00	95.00	1.11	0.29
IQ	80.50 (18.51)	78.35 (18.85)	0.36	0.72
Diagnosis			0.17	0.68
Autistic Disorder (%)	100	85	3.24	0.07
PDD-NOS (%)	0	15		
Severity of Disorder				
ADI-R Social Interaction	24.60 (4.64)	23.20 (4.48)	0.97	0.34
ADI-R Communication	18.65 (4.06)	19.20 (4.25)	−0.42	0.68
ADI-R Stereotyped Behavior	6.60 (2.60)	6.45 (2.94)	0.17	0.87
ADI-R Abnormal <36 months	3.15 (1.73)	3.60 (1.82)	−0.80	0.43

*Notes.* ADI-R = Autism Diagnostic Interview-Revised; IQ = Intelligence quotient as assessed by the Chinese version of the Wechsler Intelligence Scale for Children-Fourth Edition (Hong Kong) or the Stanford-Binet Intelligence Scale-Fourth Edition; PDD-NOS = Pervasive Developmental Disorders Not Otherwise Specified. Standard deviations are in parenthesis.

### Procedures

Prior to the baseline assessment, the children and their parents were briefed on the assessment procedure and informed consents were obtained from the parents. The assessment was conducted at the Hong Kong Polytechnic University. The children were individually assessed in a quiet room by trained research assistants on their intellectual functioning, self-control, and scalp EEG activities. During the assessment, the children’s parents were interviewed by a clinical psychologist on their developmental and medical histories using a structured clinical interview. The research assistants who conducted the neuropsychological and EEG assessments were blinded to the rationale of the study and the group assignment. During the EEG assessment, each child was required to perform an inhibitory control task (i.e., the Go/No-go task) while their EEG data were obtained using a TruScan measuring set with 19 electrodes positioned across the scalp according to the International 10–20 System [66]. Details of the Go/No-go task are elaborated in the Measures section. The electrode impedances were maintained at *≤*10 kΩ. The EEG signals were referenced to linked ears and sampled at 256 samples per second, with a high-frequency limit band pass of 30 Hz, and then fast Fourier transformed. Artifact-free EEG data were selected based on visual examination for eye movements and muscle artifacts and then captured for subsequent sLORETA analyses. The sLORETA method is a properly standardized discrete, three-dimensional (3D) distributed, linear, minimum norm inverse solution for exact and zero-error localization of the source of scalp EEG activity [55].

The baseline assessment was performed two weeks before the start of the intervention. After the baseline assessment, the two groups of children were provided with training on *Nei Yang Gong* and Progressive Muscle Relaxation, respectively, for one month. Two weeks after the intervention, the same set of assessments on self-control, daily behaviors and EEG activity were administered to the children and their parents.

### Measures

#### Neuropsychological assessments on self-control

The children’s ability to exercise self-control was assessed by the following three standardized neuropsychological tests, namely the Tower of London Test, the Children’s Color Trails Test and the Five Point Test:

1. The Tower of London Test – Drexel Version (TOLDX) [67] is designed to test the well-controlled and flexible execution of goal-directed behavior. It consists of ten items that involve moving three colored beads on three vertical pegs to match a target arrangement while adhering to the testing rules. The test is suitable for use with children aged 7 years and above [67], although it has been used in studies with children aged as young as 4 [68]. The recipient’s self-control is measured by the total number of rule violations, with fewer violations indicating better self-control. The degree of impulsivity is indicated by the initiation time, which is measured in terms of seconds, with a longer duration reflecting less impulsivity.
2. The second trial of the Children’s Color Trails Test (CCTT-T2) [69] was adopted to test inhibitory control and flexibility in shifting mental processes and motor responses. The CCTT is designed for children aged 8 to 16 years, although some normative data are available for children aged 5 to 7 years [70]. The test involves sets of duplicate numbers embedded within pink and yellow circles, and requires the child to connect the numbers in ascending order from 1 to 15 while alternating between the two colors as quickly as possible. The completion time (in seconds) is used as a measure of inhibitory and flexible control, with a shorter duration reflecting better control.
3. The Five Point Test (FPT) [71] is a figural fluency test that was adopted to test flexible mental control, as it requires the spontaneous generation of novel designs without repetition by connecting five points with straight lines within 5 minutes. The test is suitable for children aged 6 and above [70]. The total number of unique designs generated is measured, with higher amounts indicating better self-control and greater flexibility.

#### Parental evaluation on behavioral measures

The parents were interviewed on their child’s behavioral changes in daily life using standardized questionnaires. As problems with self-control have been found to be associated with typical autistic symptoms (e.g., repetitive/disinhibitory behaviors, communication and socialization problems), the parents were asked to rate their child’s autistic symptoms based on the Autism Treatment Evaluation Checklist [72] before and after the intervention. The ATEC was selected because it is a simple one-page evaluation that has been empirically found to be a sensitive measure for evaluating treatment effectiveness for individuals with ASD [73]–[75]. Moreover, some preliminary evidence suggests that it is able to monitor the progress of children with ASD with high internal consistency [76]. The ATEC consists of four subscales: (1) speech/language communication; (2) sociability; (3) sensory/cognitive awareness; and (4) health/physical behavior. The total scores of the four subscales were used for the pre-post comparison of each group.

The parents were also asked to answer another questionnaire containing items about their child’s ability to exercise self-control, including the control of his/her temper tantrums and rigid thoughts/acts, and their difficulty in verbal expression. This questionnaire was used in our previous study to evaluate the effect of the *Chan*-based diet modification in improving the self-control of children with ASD [23]. The parents’ rating on this questionnaire was consistent with their rating on the standardized ATEC. Given that the rationale of the present study was to examine the treatment effect on self-control, it was anticipated that the children would show improvement in self-control, but not in verbal expression. The questionnaire asked the parents to evaluate the degree of change in each problem behavior after the one-month intervention on a scale of “−5” to “+5”, where “−5” indicates a “large reduction in problem behavior”, thereby indicating a large degree of improvement; “+5” indicates a “large increase in problem behavior”, thereby indicating a large decline; and “0” indicates “no change”. The mean rating obtained from each group was used for the subsequent between-group comparisons.

#### Event-related EEG assessment

The event-related EEG signals for each child were collected during an inhibitory control test, namely, the Go/No-Go task. The Go/No-Go task is a computerized test that measures the respondent’s ability to flexibly respond to changing stimuli and inhibit unwanted responses. A total of 192 black balls and 48 red balls (black:red ratio = 4∶1) were randomly displayed, one at a time, for 500 ms followed by 1000 ms of blank intervals, in the center of a computer screen. The child was required to press a key as quickly as possible in response to a black ball (Go stimulus), but to inhibit their response when a red ball (No-go stimulus) appeared. The total testing time was 6 minutes. As the No-go condition required the inhibition of an unwanted response (i.e., pressing the key), the EEG data collected during this condition were selected for subsequent sLORETA analysis of the inhibition-related neural activity.

The EEG data for each child was first transformed using Excel before being imported into EEGLAB software using MatLab 7.1 to capture the correct events and epochs. The epoch limits were set as 50ms as the start and 900 ms as the end. Artifacts in the epoched data were then pruned by visual inspection and the rejection method on the EEG Plot. The selected data were exported and then spectrally processed by fast Fourier transformation (FFT) for computation of the power data in the theta band (4–7.5 Hz) through the use of the NeuroGuide software. As the ACC has been reported to be one of the generators of theta activities in the human brain [77]–[79] and to play a major role in inhibitory control, sLORETA [55] was thus adopted to localize the sources of the theta activities in response to the “No-go” condition. The sources of the theta activities were expressed as the three-dimensional cortical current density according to the Montreal Neurological Institute (MNI) brain coordinates.

### Intervention: *Nei Yang Gong* vs. Progressive Muscle Relaxation

The children in the experimental and control groups attended training classes at the Chinese University of Hong Kong twice per week for four weeks, with each session lasting for an hour. The two training classes were administered on the same days of a week.

The children in the experimental group were taught to practice *Nei Yang Gong* by a clinical psychologist, with over 10 years clinical experience in training children with autism. The *Nei Yang Gong* set comprised five types of movement: tranquil stand, shoulder relaxation, nasal bridge massage, *Qi*-circulating movement, and passive *Dan Tian* breathing. The movements were arranged in a fixed sequence and incorporated with specific pieces of music to facilitate the children’s mastery of the technique and to keep them engaged. While practicing *Nei Yang Gong*, the children were guided to move calmly and relaxingly, and were encouraged to persist with the movements. To foster self-awareness and self-control, the children were also encouraged to practice some forms of *Nei Yang Gong* that served as self-guided massages for relaxing and calming oneself whenever they feel distressed and frustrated, e.g., rolling their hands slowly up and down between the chest and the abdomen, resting their hands on their abdomen while quietly observing their breathing. The selected *Nei Yang Gong* movements involved simple bodily actions (e.g., moving hands/fingers up and down, and bending the knees) and the children were only asked to perform the movements in a relaxed and natural manner. Five years of clinical observation suggested to the research team that even children with moderate grade mental retardation would be able to learn and practice *Nei Yang Gong*. Each child was closely monitored by the clinical psychologist during each session to ensure that all the children were able to master the technique. As the intervention progressed, the children were observed to become less agitated and to move at a slower pace when practicing *Nei Yang Gong*.

Each round of *Nei Yang Gong* with music lasted for 5 minutes. The children were encouraged to practice the movements daily one to three times. The children’s practicing times and frequencies were recorded in log booklets, which indicated that 65% of the children practiced *Nei Yang Gong* at least 6 days per week for between 5 and 45 minutes a day (mean = 19.38; SD = 11.82). The remaining children practiced 5 to 25 minutes a day, 3 to 5 days per week (mean = 13.66; SD = 6.12). Given that the physical conditions vary among children, they were instructed to stop doing the exercise until they began sweating to avoid exhaustion. Thus, the duration of practice was not fixed.

The children in the control group were taught the Progressive Muscle Relaxation (PMR) technique by another clinical psychologist with over a decade of experience in administering PMR to pediatric population. The Chinese version of PMR for children was adopted. This version was locally developed by the Clinical Psychology Division of the Hong Kong Psychological Association, and has been used in pediatric clinical health since 2004. Clinical experience and empirical evidence [50], [51] suggested that even children with mental retardation would be able to master the PMR technique. In each training session, the therapist guided the children in sequentially tensing and relaxing seven muscle groups (nose, mouth, shoulders, arms, hands, chest and feet) as instructed by a sound track. To facilitate the learning of the ASD children, during the practice, visual and verbal cues were provided on how to contract and relax each muscle group (e.g, the tensing-relaxing of the arm muscle was represented by an image of a monkey swinging from one tree to another printed on a cue card together with the voice of a monkey presented on the sound track). The therapist monitored the progress of each child and observed that all children were able to master the technique. Each round of PMR lasted about 20 minutes. In addition to the PMR technique, other behavioral-based training techniques on social skills, emotional awareness and behavioral monitoring were incorporated into the training program.

After the first training session, the children were given a cue card that listed the seven steps of the PMR technique for home practice. In each subsequent training session, any difficulties the children encountered during their home practice were reviewed. Half the children practiced PMR once (i.e. 20 minutes) a day at least 6 days a week, while the other half practiced between 1 and 5 days per week. The average practice duration of the control and experimental groups was similar, *t* = −1.28, *p* = 0.21.

### Data Analyses

The mean performance of each group on the self-control neuropsychological test measures and the mean parental rating on the ATEC at pre- and post-training were compared using repeated measures ANOVAs and then followed by *post hoc* paired sample *t* tests. The change in mean performance/rating in each group was also computed for between-group comparison using independent sample *t* tests. To examine the effects of the two types of training on neural activity, the regions of interest (ROI) analysis of the sLORETA method was used to localize the source of the scalp-EEG activity and to compare within and between groups using voxel-by-voxel paired sample *t* tests and independent sample *t* tests respectively. The sLORETA comparisons were computed with subject-wise normalization and log transformation for each group to compare the pre-post changes in the sources of theta activity in the ACC during the “No-go” condition. Because specific hypotheses were tested, no adjustment to the alpha level was applied for the planned comparison to avoid lowering the power of the tests.

## Results

### *Nei Yang Gong* Improves the Self-Control Test Performance

Repeated measures ANOVAs were performed separately for each neuropsychological test measures to compare the effects of *Nei Yang Gong* and PMR in enhancing the self-control of ASD children. At the baseline, the levels of self-control in both groups were comparable, as reflected by the three neuropsychological test measures, which showed *t* ranges from −1.49 to 1.5, and *p* ranges from 0.15 to 0.72. The results of the repeated measures ANOVAs showed that there were significant and marginally significant Time (Pre vs Post) by Group (Control vs Experimental) interaction effects on the two indices of the TOL^DX^, i.e., the frequency of rule violation, *F*(1,34) = 6.02, *p* = 0.02, and the initial time, *F*(1,34) = 3.25, *p* = 0.08. For the other two measures, there was a significant main effect of Time (CCTT-T2: *F*(1,33) = 13.23, *p* = 0.001, FPT: *F*(1,35) = 18.05, *p* = 0.00).

Subsequent post hoc *t* tests confirmed that the four neuropsychological test measures were consistent, indicating that the autistic children in the experimental group showed better self-control than those in the control group after the one-month intervention (see Table 2). Although both groups showed improvement in reducing the frequency of rule violation in the TOL^DX^, the mean reduction of the experimental group (−8.53) after the intervention was about four times that of the control group (−2.82), and the group difference was statistically significant with a large effect size (0.84), *t*(34) = 2.45, *p* = 0.019. Furthermore, the experimental group, but not the control group, became less impulsive in problem-solving, as reflected by their significantly increased average initial time in attempting the TOL^DX^ questions [experimental: *t*(18) = −3.65, *p* = 0.002, effect size = 0.86; control: *t*(16) = −0.58, *p* = 0.57, effect size = 0.14]. The reduction in impulsivity of the treatment group was greater than that of the control group, with a medium effect size (0.77), *t*(33) = −2.27, *p* = 0.03. In regard to the CCTT-T2, the children in the experimental group showed a significant reduction in completion time (mean reduction = −17.29) with a large effect size (0.83), *t*(17) = 3.53, *p* = 0.003, while the children in the control group did not show any significant improvement (mean reduction = −6.97), *t*(16) = 1.71, *p* = 0.11, effect size = 0.41. Although the between-group difference in the mean reduction of completion time did not reach statistical significance, *t*(33) = 1.61, *p* = 0.12, the effect size (0.55) is at the medium level. Similarly, children in both the control group and the experimental group generated significantly greater numbers of unique designs in the FPT after the intervention (mean increment: control group = 4.11 and experimental group = 5.11), *t*(17) = −2.69, *p* = 0.015 and *t*(18) = 3.50, *p* = 0.003, respectively. Yet, the change in the experimental group was relatively more robust (a large effect size of 0.80) than that in the control group (a medium effect size of 0.63). In sum, the children who received training in *Nei Yang Gong* showed more noticeable improvement in self-control than the children in the control group across all the neuropsychological tests.

**Table 2:** Mean Performance and Difference Score across the Neuropsychological Tests on Self-Control of the Control and Experimental Groups at Pre- and Post-One-Month Intervention.

	Control Group			Effect	95% C.I.	*p* value	Experimental Group			Effect	95% C.I.	*p* value
	(n = 19)			Size			(n = 20)			Size		
	Pre	Post	Diff				Pre	Post	Diff			
TOL^DX#^												
Rule Violation	3.88	1.06	−2.82	1.11++	1.52–4.13	0.00**	14.37	5.84	−8.53	0.92++	4.06–12.99	0.00**
	(3.30)	(2.16)	(2.49)				(13.22)	(7.07)	(9.27)			
Initial Time	15.48	16.10	0.62	0.14	−2.89–1.65	0.57	10.73	14.92	4.19	0.86++	−6.62 – −1.77	0.00**
	(12.29)	(9.36)	(4.41)				(5.36)	(6.56)	(4.87)			
CCTT-T2#												
Completion Time	59.38	52.41	−6.97	0.41	−1.68–15.63	0.11	76.61	59.32	−17.29	0.83++	6.95–27.63	0.00**
	(24.76)	(21.71)	(16.83)				(41.22)	(34.26)	(20.79)			
FPT												
Unique Designs	15.33	19.44	4.11	0.63+	−7.33 – −0.89	0.02*	14.05	19.16	5.11	0.80++	−8.17 – −2.04	0.00**
	(12.43)	(11.67)	(6.48)				(8.63)	(10.31)	(6.37)			

*Note.* C.I. = Confidence Interval; Diff = average of difference score by subtracting pre-training score from post-training score; TOL^DX^ = The Tower of London Test – Drexel Version; CCTT-T2 = Trial 2 of the Children’s Color Trails Test; FPT = Five Point Test. Standard deviations are in parenthesis.

# Lower value indicates better performance;

* *p*<0.05,

** *p*<0.01;

++ large effect size,

+ medium effect size.

### *Nei Yang Gong* Reduces Self-Control Related Daily Behavioral Problems

While the children who learned *Nei Yang Gong* demonstrated better performance in the laboratory tests of self-control, we then further analyzed their ability to exercise self-control in everyday life. The parents were interviewed using the ATEC on the common autistic symptoms before and after the intervention, and were given a questionnaire rating the treatment effect in controlling temper and obsessive behaviors, and verbal expression difficulty after intervention.

The results of the repeated measures ANOVAs showed that there were significant main effects of Time on the sociability subscale, *F*(1,37) = 14.25, *p* = 0.001, and the health/physical/behavior subscale, *F*(1,37) = 4.74, *p* = 0.04, and a marginally significant main effect of Time on the sensory/cognitive awareness subscale, *F*(1,37) = 3.20, *p* = 0.08. Given the significant main effects of the three measures, the post hoc *t* statistics are provided to shed some light on the possible pre-post changes between the groups. Table 3 presents the pre-post comparisons on the subscales of the ATEC. The baseline level of the mean rating across all subscales is comparable between the two groups, with *t* ranging from 0.68 to 1.48, and *p* ranging from 0.15 to 0.50. After intervention, parents of the experimental group reported significant improvement in three subscales: sociability, *t*(19) = 3.06, *p* = 0.006, effect size = 0.68; sensory/cognitive awareness, *t*(18) = 2.11, *p* = 0.049, effect size = 0.49; and health/physical/behavior, *t*(18) = 2.87, *p* = 0.01, effect size = 0.66. However, the children in the control group were rated as showing significant improvement mainly in the sociability subscale, *t*(18) = 2.53, *p* = 0.02, effect size = 0.58. As expected, as the two intervention programs aimed to improve self-control, neither groups showed any change in the speech/language/communication subscale, as revealed by the non-significant results of the *F* test, *F*(1,37) = 0.01, *p* = 0.92. These results suggest that the mind-body exercise had a positive effect on the cognition and health of children with ASD, which may not be achieved by conventional behavioral intervention.

**Table 3:** Mean Parental Rating on the Autism Treatment Evaluation Checklist (ATEC) of the Control and Experimental Groups at Pre- and Post-One-Month Intervention.

	Control Group		Effect	95% C.I.	*p*	Experimental Group		Effect	95% C.I.	*p*
	(n = 19)		Size		value	(n = 20)		Size		value
	Pre	Post				Pre	Post			
Speech/Language/	6.53	5.68	0.27	−0.70–2.39	0.27	5.80	5.05	0.29	−0.46–1.96	0.21
Communication	(3.39)	(2.43)				(3.25)	(2.65)			
Sociability	17.53	14.63	0.58+	0.49–5.30	0.02*	15.55	13.50	0.68+	0.65–3.45	0.01*
	(6.28)	(6.68)				(6.18)	(5.91)			
Sensory/Cognitive	12.32	11.58	0.20	−1.06–2.53	0.40	10.63	9.21	0.49	0.01–2.83	0.05*
Awareness	(5.28)	(5.37)				(5.51)	(4.91)			
Health/Physical/	19.16	17.11	0.26	−1.72–5.83	0.27	15.00	12.26	0.66+	0.73–4.74	0.01*
Behavior	(9.46)	(11.89)				(7.78)	(6.29)			

*Note.* C.I. = Confidence Interval; Standard deviations are in parenthesis. Lower value indicates less severe problems;

* *p*<0.05,

** *p*<0.01;

+ medium effect size.

**Figure 2:**
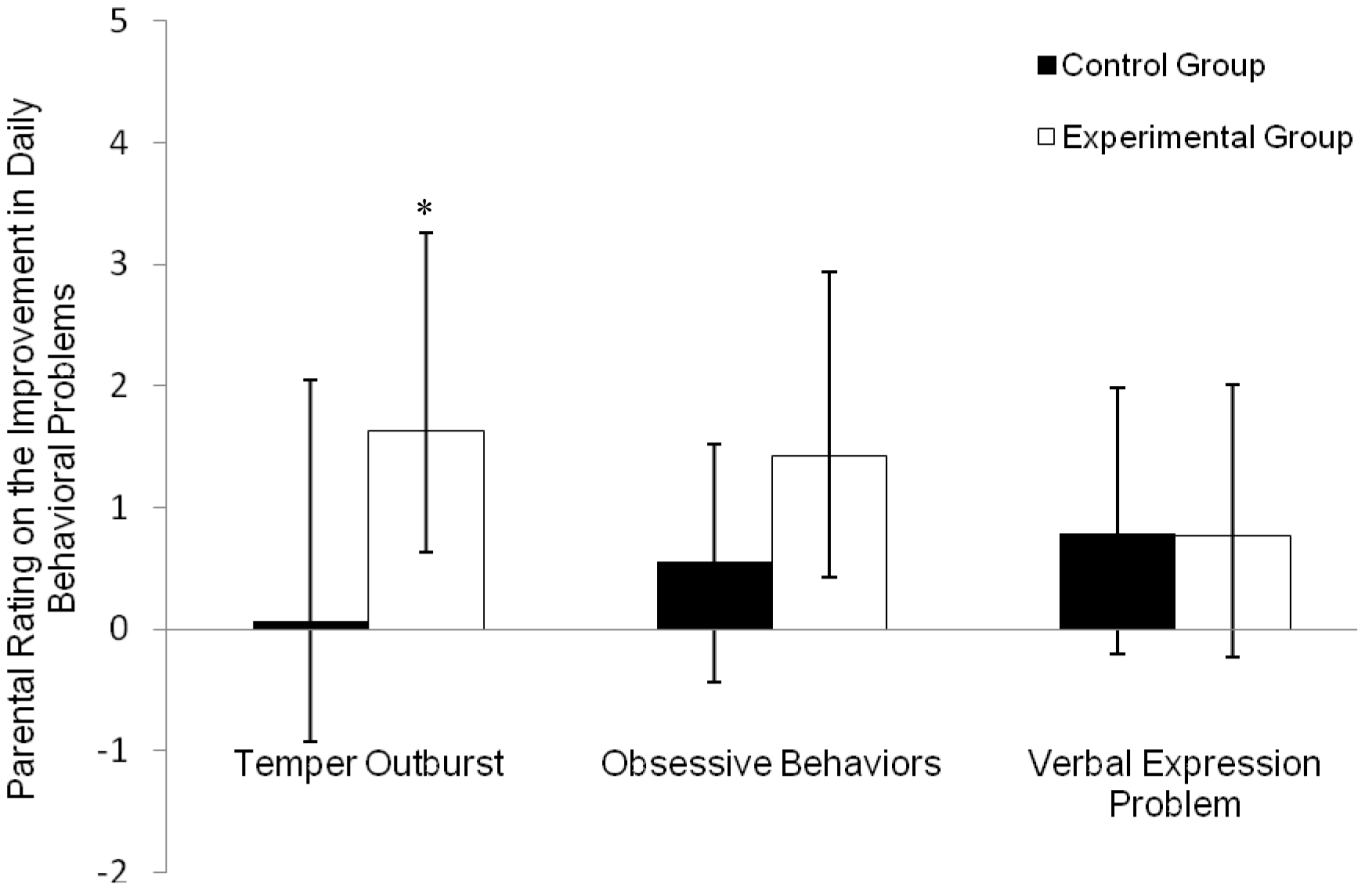
Parental rating of the treatment effects after one-month training. The treatment effects as rated by the parents in controlling the children’s temper outburst and obsessive behaviors, and verbal expression problem in daily life after one-month training. A positive value indicates improvement in the corresponding problem. * *p*<0.05 (independent sample *t* test).

The results of the parents’ evaluation of the changes in their child’s temper outbursts, obsessive behaviors and verbal expression problem, are also consistent with the above-mentioned findings. With the temper outburst scale, the experimental group reported a significantly greater reduction (−1.63) than the control group (−0.07), with a large effect size (0.86), *t*(28) = 2.36, *p* = 0.026 (see Fig. 2). With the obsession scale, the experimental group reported a mean reduction of 1.43 and the control group a reduction of 0.56, while difference was only marginally significant, *t*(28) = 1.90, *p* = 0.068, it had a medium effect size (0.69). With the verbal expression problem subscale, the experimental (−0.77) and control (−0.79) groups both showed no obvious change, *t*(28) = 0.35, *p* = 0.97, effect size = 0.02. Thus, the parents’ reports are consistent with the neuropsychological assessments that indicate that the mind-body exercise appeared to have some positive effects in improving the self-control of the ASD children. As expected, the children did not show any improvement in language expression as it was not the target of the training exercises. Again, the parental reports on this specific treatment effect cohered with the neuropsychological test results and our anticipated results, suggesting that the parents’ ratings were not biased (e.g., reporting exaggerated positive effects).

In addition to the positive effects, no parent reported any adverse effect of the two interventions during the study. Although some children may have experienced fatigue while practicing *Nei Yang Gong*, they were advised to stop whenever they felt tired or discomfort, and to resume practice after taking a rest.

### *Nei Yang Gong* Enhances Brain Activity in the Anterior Cingulate Cortex

**Figure 3:**
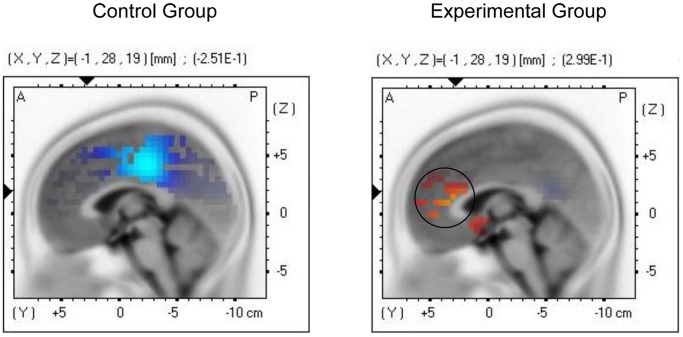
Neuro-electrophysiological activity changes after one-month training. Graphical representation of the sLORETA paired *t*-statistics results comparing the pre- and post-one-month theta source activity of the control and experimental groups during the No-go condition of the Go/No-go task. The regions colored in red indicate significantly elevated ACC activity (in the circle) after one-month *Nei Yang Gong* training (experimental group) at *p<*0.05, which was not observed after practicing the Progressive Muscle Relaxation (control group).

Further pre-post comparisons of the EEG activity of the groups during the Go/No-go task (a common test of inhibitory control) were performed using the sLORETA method. The No-go condition, which requires the inhibition of a behavioral response, was selected for analysis. The ACC was set as the region of interest because it has been found to mediate inhibitory control [56]–[58] and to be hypo-active in individuals with ASD [59]–[61]. The effects of *Nei Yang Gong* and PMR in enhancing ACC activity during the No-go condition were compared using the sLORETA voxel-by-voxel paired *t* statistics. At the baseline, the results of the voxel-by-voxel independent *t* statistics show comparable ACC activity levels between the two groups during the No-go condition, *t* = 0.76, *p = *0.45. After one-month intervention, the experimental group showed significantly elevated activity in the rostral ACC region (Brodmann areas 24) during the No-go condition, *t = *0.30, *p = *0.02 (circled region in the right-side image in Fig. 3). Treatment-induced activity elevation in this ACC region has been previously found to indicate the level of hypoactivity in children with ASD as compared with normally developed children [60]. In contrast, the control group did not show any significant change in activity in the ACC, *t* = −0.25, *p = *0.81 (left-side image in Fig. 3). The enhanced ACC activity in the experimental group indicates that the ACC may be the neural mechanism underlying the positive treatment effects of *Nei Yang Gong* on the self-control of ASD children.

## Discussion

Self-control problems, such as emotional outburst, repetitive or obsessive thoughts or acts, and impulsivity, are common behavioral manifestations in children with ASD. These problems hinder learning and social development, and increase caregiver burden. The present findings supported the positive effects of the Chinese *Chan*-based mind-body exercise, *Nei Yang Gong*, in enhancing self-control and reducing the typical autistic symptoms and daily emotional and behavioral problems of children with ASD. A one-month *Nei Yang Gong* training program, totaling 8 one-hour sessions, was able to elicit robust treatment effects that were not observable for the other group receiving a conventional behavioral training technique. One encouraging finding of the present study is that this exercise can be mastered by children with limited language ability and intellectual functioning. Because of the simplicity and effectiveness of this Chinese mind-body exercise, it can be considered as an alternative or complementary intervention for improving executive control in children with ASD.

The potential benefits of the mind-body exercise in promoting increased self-control found in the present study are consistent with previous empirical studies on mind-body interventions. For instance, Bahrami et al. [31] reported a 42% reduction in stereotypic behavior in ASD children after 14 weeks of Kata techniques (a form of mind-body exercise) training compared to the no-exercise control group, which was sustained at one-month follow-up. Another study [37] also reported significantly reduced behavioral problems among 24 children with ASD after 8-weeks movement-based therapy (with yoga as one of the treatment components). A recent review article in *Science* [80] concluded that traditional martial arts (a form of mind-body exercise) improved executive control to a greater extent than standard physical education [36] or modern martial arts [81] for children/adolescents with executive function deficits. The effectiveness of the traditional martial arts may be related to their concurrent emphasis on physical training, self-control, discipline, and character development (e.g., respect, responsibility, and perseverance), which tend to focus more on the executive function. Similarly, as the Chinese mind-body exercise adopted in the present study was developed based upon the principles of Chinese *Chan* and Buddhism, the children were guided to practice with a peaceful mind and to relieve anger and distress. This may explain the distinctive effects of the exercise in enhancing self-control.

The underlying mechanism that may explain the change in the participants’ self-control and daily behaviors was indicated by the change in EEG activity as analyzed by the sLORETA method. The findings show that the children who practiced *Nei Yang Gong* for one month had significantly increased electrophysiological activity in the rostral ACC region, whereas those who practiced PMR did not show such elevation. Studies have been repeatedly reported that children with ASD show hypoactivity in the ACC during response-monitoring and inhibitory tasks (e.g., the Go/No-go task) as compared to normally developed children [59]–[61]. The enhanced ACC activity while performing an inhibitory task observed among the children who completed one-month *Nei Yang Gong* practice suggests that the behavioral improvement of children with ASD may be associated with increased ACC activity. In fact, the effect of *Chan*-based mind-body exercise on the brain was explored in our previous randomized controlled study [43]. This study found that individuals who practiced mind-body exercises were able to foster a relaxed and attentive brain state as reflected by their altered EEG indices, whereas those practicing PMR did not show any alteration in brain activity. A relaxed and attentive brain state is crucial for achieving peak performance and exercising good self-control. Nevertheless, how mind-body exercises change and increase the activity in the neural system warrants further investigation.

The therapeutic effects of *Nei Yang Gong* on brain functions and activity observed in the present study are in line with our other clinical observations and empirical findings in recent years. For instance, two case studies, one on a child with autism and mental retardation and another on an adolescent with Asperger’s disorder, reported significantly improved inhibitory control of emotional and behavioral disturbances after DMBI, with *Nei Yang Gong* as one of the treatment components [22], [24]. A recent randomized controlled study of the *Chan*-based mind-body intervention through dietary modification on children with ASD also showed significant enhancement in inhibitory control and elevation in ACC activity after one-month treatment [23]. Other positive outcomes on emotional control, cognitive functions (e.g., attention), and frontal brain activity were also observed in three randomized controlled trials on patients with major depressive disorders and community-dwelling adults after DMBI [25]–[26], [40]. Thus, the results of the present study shed further light on the potential application of Chinese *Chan*-based mind-body exercise as a complementary intervention for the rehabilitation of individuals with ASD and other emotional and cognitive problems.

The results of the present study indicate that the one-month Chinese mind-body exercise had positive effects in improving the self-control and alleviating the autistic symptoms of children with ASD. However, the long-term effects of the exercise and the sustainability of the treatment effects remain unknown and are worth further investigation. This study also explored the treatment effect of a single component (mind-body exercise) of the DMBI. Our previous study reported a similar positive outcome for another component (diet modification) of the DMBI in improving the self-control of children with ASD [23]. Thus, future studies could investigate whether the use of both treatment components could have additive treatment outcomes when applied to individuals with ASD. Because self-control problems are commonly observed in patients with other brain disorders (e.g., attention-deficit/hyperactivity disorder, traumatic brain injury, and dementia), it may also be worth extending the investigation of the effects of mind-body exercise to these clinical populations. Last but not least, given the increasing research interest in mind-body interventions (e.g., yoga) for improving health in Western countries, future studies could also investigate the applicability and effectiveness of this Chinese *Chan*-based mind-body exercise in Caucasian populations.

## Conclusions

In summary, this study provides evidence that one month of training in the Chinese *Chan*-based mind-body exercise, *Nei Yang Gong*, had a positive effect in enhancing the self-control of children with autism spectrum disorders. This cognitive enhancement coincided with significantly elevated brain activity in the anterior cingulate cortex of the *Nei Yang Gong* group participants. In contrast, this improved brain functioning and elevated brain activity was not observed in the children practicing progressive muscle relaxation. This encouraging finding confirms the potential clinical applicability of this Chinese mind-body exercise in enhancing the self-control of individuals with various brain disorders.

## Supporting Information

Checklist S1 CONSORT checklist.
(DOC)

Protocol S1 Trial protocol.
(DOC)
