# Supplementary material for: A Chinese Mind-Body Exercise Improves Self-Control of Children with Autism: A Randomized Controlled Trial
Source: PLoS One. 2013 Jul 10;8(7):e68184. doi: 10.1371/journal.pone.0068184 (PMC3707921; doi:10.1371/journal.pone.0068184)
Supplement: Protocol S1 — Trial protocol. (DOC) [file pone.0068184.s002.doc]

**Effects of Mind-Body Intervention on Improving Cognitive Functions and Behaviors of Children with Brain Dysfunction**

**Principal Investigator**

**Dr. Mei-chun CHEUNG (ITC/POLYU)**

**Co-Investigators**

**Prof. Agnes S. CHAN (PSY/CUHK)**

**Dr. Sophia L. SZE (PSY/CUHK)**

**Dr. Yvonne M. HAN (SEN/HKIEd)**

SUMMARY

Brain dysfunction refers to abnormal cognitive, emotional and behavioral problems due to brain disorders. Brain dysfunction can arise as a result of developmental factors, illnesses, or accidents. Common developmental brain disorders include autism, attention-deficit/hyperactivity disorder (ADHD) and Down's Syndrome. Acquired brain disorders include brain injury related to car accidents, or illnesses such as epilepsy and stroke. As of today, there is no effective pharmacological intervention to improve the cognitive and behavioral problems of patients with brain disorders.

The Chinese *Chan*-based Mind-Body Intervention (namely *Dejian* Mind-body Intervention, DMBI) is a newly developed mind-body intervention based on Chinese *Shaolin* *Chan* (or Zen) principles. Cumulative empirical evidence in past five years has revealed preliminary support for its positive effects of DMBI on cognitive functions, mood state, behaviors and / or neurophysiological activities among individuals with autism, Asperger’s disorder, severe brain damage, major depressive disorders, and community-dwelling adults. These research findings are in line with over a hundred years of clinical observation of improved mental and physical health conditions of patients with various brain dysfunctions, psychiatric illnesses, and physical illnesses.

Given some empirical support for the positive effects of DMBI for patients with brain disorders, the present study thus aims to further investigate the therapeutic effect of DMBI on improving cognitive abilities and problematic behaviors of children with brain dysfunctions, in comparison with that of conventional cognitive and / or behavioral treatment which has been found to be effective in improving the behavioral problems and cognitive abilities of patients with brain disorder.

A total of 80 children and adolescents (aged 6 to 17) with neurological disorders that may include autism, ADHD, epilepsy, or stroke (the final patient group will be determined according to accessibility) that have cognitive and behavioral dysfunction will be recruited for this study. Half of the patients will be randomly assigned to the group receiving DMBI, while the other half will be receiving conventional cognitive and / or behavioral treatment. Both intervention groups will be administered twice per week, for four weeks. Changes in their cognitive functioning, behaviors and brain activities will be measured before and after the one-month intervention, and compared between the two groups. The results will provide evidence to show whether DMBI is effective in improving functional level and neurophysiological status of patients with brain disorders, and if so the possibility of applying Chinese *Chan* health concepts as neuropsychological intervention on other disorders may be explored.

**Effects of Mind-Body Intervention on Improving Cognitive Functions and Behaviors of Children with Brain Dysfunction**

**1. OBJECTIVES**

Given the encouraging findings from clinical observations and empirical evidence for the positive effect of DMBI on different brain disorders, the present study aims to investigate the effect of the DMBI on improving cognitive and psychosocial functioning of patients with brain disorders, and to explore the possibility to apply Chinese *Chan* health concepts as neuropsychological intervention.

The purposes of the study are:

1. To compare the effect of DMBI with that of conventional cognitive / behavioral therapy on improving cognitive functions and reducing behavioral problems of patients with brain disorders.
2. To utilize eletro-encephaolography (EEG) method to study the neurocognitive basis and the underlying nature of the therapeutic effect of the DMBI.
3. To explore the possibility to apply Chinese *Chan* health concepts as neuropsychological intervention.

**2. BACKGROUND OF RESEARCH**

1. Work Done by Others

*Mind-body intervention*

Mind-body intervention emphasizes the interaction among the brain, the mind, and the body. The concept that the mind plays an important role in the improvement of health is a core component of traditional Chinese medicine that dated back at least two thousand years in China. With the emphasis that treatment should be holistic in nature that includes moral, spiritual and environmental factors, Chinese have developed different kinds of mind-body intervention including *qigong*, *tai chi*, relaxation training, thought training (e.g., *Chan* or Zen), and dietary modification.

There has been scientific and clinical studies which demonstrated the therapeutic effects of mind-body intervention in clinical and normal populations (see Klein & Adams, 2004 for review). In clinical practice, an increasing number of empirical studies have reported positive effects of mind-body training on different mental and physical problems, such as depression (Nakao et al., 2001; Tsang, Cheung, & Lak, 2002), anxiety (Deckro et al., 2002), obsessive-compulsive disorder (Nakao et al., 2001; Schwartz & Begley, 2002), insomnia (Jacobs, Benson, & Friedman, 1993), chronic pain (Caudill, Schnable, Zuttermeister, Benson, & Friedman, 1991), hypertension (Benson, Rosner, Marzetta, & Klemchuk, 1974), and cardiac diseases (Leserman, Stuart, Mamish, & Benson, 1989). Apart from encouraging effects on mental (see review, Jacobs, 2001; Mandle, Jacobs, Arcari, & Domar, 1996) and physical health (Lai, Lan, & Wong, Teng, 1995; Lan, Chen, Lai, & Wong, 1999; Yeh et al., 2004; Young, Lawrence, Jee, & Miller, 1999) in clinical samples, some empirical data have also suggested that mind-body training has a positive impact on cognitive, emotional and psychological problems in normal population (Chan et al., 2007; Chou et al., 2004; Jin, 1989; Jin, 1992; Li, Duncan, & Duncan, 2001; Li, McAuley, Harmer, Duncan, & Chaumeton, 2001; Sandlund & Norlander, 2000; Wang, Taylor, Pearl, & Chang, 2004). It can be seen that the therapeutic effect of mind-body intervention have begun to be evidenced by empirical studies.

*Cognitive-Behavioral Therapy (CBT) or Behavioral Intervention*

The Cognitive-Behavioral Therapy (CBT) and Behavioral Therapy are well-established and commonly practiced psychological interventions that have been found to be effective for treating a variety of conditions within the pediatric populations, including mood, anxiety, anger management, disruptive or repetitive behaviors, and cognitive dysfunctions. For instances, CBT has been applied to and found to have positive effects on improving mood or reducing anxiety of children with autism spectrum disorders (Lang, Regester, Lauderdale, Ashbaugh, & Haring, 2010; Moree & Davis III, 2010), depression, and anxiety disorders (Compton, March, Brent, Albano, Weersin, & Curry, 2004). Empirical evidence has also supported the effect of behavioral-based interventions in improving language, cognitive abilities, adaptive behavior, social skills, and reducing aggression of children with autism (Dawson & Burner, 2011), improving self-control of children with Tourette disorder (Piacentini, Woods, Scahill, et al., 2010), improving attention and inhibitory control of behaviors in children with ADHD (Waxmonsky, Waschbusch, Pelham, Draganac-Cardona, Rotella, & Ryan, 2010).

(B) Work Done by Us

*Effect of Chinese Mind-body intervention on Cognitive Functioning*

The authors’ group has been exploring the effect of different Chinese mind-body intervention in promoting the cognitive functioning in adults and children. In one study, the first Co-Investigator has developed a set of easy-to-practice, mind-body intervention for children, based on traditional Chinese medicine concept, and have conducted a study using this 40-session intervention for primary school children with behavioral problems (Chan, Cheung, & Sze, 2007). Results indicate that, compared with controls who underwent remedial classes, children of the intervention group showed a significant reduction in withdrawn and attention problems. They also showed improvements in cognitive functioning, including memory and cognitive flexibility. In another study (Chan, Han, & Cheung, 2008), our group examined brain activities as measured by EEG when normal healthy young adults practiced a traditional Chinese relaxation method, the Triarchic Body Pathway Relaxation Technique. Results indicated increased left-sided brain activation and frontal midline power, suggesting that the mind-body training gives rise to positive emotional experience and focused internalized attention. In a third study comparing Chinese mind-body exercise and cardiovascular exercises in older adults (Chan, Ho, Cheung, Albert, Chiu, & Lam, 2005), our group found that those who practiced traditional Chinese mind-body exercise or cardiovascular exercise had significantly better memory functioning than those who did not practice any exercise, and the two exercises appear to have a synergistic effect in helping to preserve memory in older adults.

*Dejian Mind-body Intervention (DMBI)*

A recent direction of research of our group is on the DMBI. Encouraging findings have been cumulated in past five years of empirical studies on the health-promoting effect of the DMBI.

*On children with autism spectrum disorder (ASD)*. A recent randomized controlled study (Chan, Sze, Han, & Cheung, In Press) has reported the positive effect of a one-month Chinese *Chan*-based dietary modification on enhancing mental flexibility and inhibitory control of children with ASD. Their cognitive function enhancement was found to be in line with their reduced repetitive/disinhibited behaviors and social deficits and their enhanced event-related EEG activity at the anterior cingulate cortex (a region mediates inhibitory control). In contrast, the control group which has no change in diet did not show any significant improvements after one month.

*On adults with depressive mood and patients with clinical depression*. In another randomized controlled study (Chan, Cheung, Tsui, Sze, & Shi, 2011), 40 community-dwelling adults with various degree of depressive mood were randomly assigned to the experimental (DMBI) and control (Cognitive-Behavioral Therapy; CBT) group to undergo a total of four ninety-minute weekly sessions of the respective intervention. Results indicated that while among individuals with moderate to severe depressive mood at baseline, only those in the DMBI but not the CBT group showed significant reduction in depressive mood, as measured by the Chinese Beck Depression Inventory. In addition, only the DMBI group demonstrated a significant increase in prefrontal activation asymmetry, suggesting increase in positive affect. Similar therapeutic effects of DMBI were found on a group of patients with major depressive disorders in two randomized controlled studies (Chan, Wong, Sze, Kwong, Han, & Cheung, 2012; Chan, Wong, Sze, Kwong, Han, & Cheung, In Press), where 75 patients with major depressive disorders were randomly assigned to receive either the DMBI or the CBT, or to the waitlist control group. The pre- and post-10-week assessment results showed that the DMBI group exhibited significantly greater extent of improvement in overall sleep quality, depressive symptoms, concentration ability and gastrointestinal health than those receiving CBT or on waitlist. Also, more participants in the DMBI group reported reduced intake of antidepressants than the other two groups.

*On single cases of epilepsy, autism, or Asperger’s syndrome*. Three case studies on chronic epilepsy (Chan, Sze, Cheung, Lam, & Shi, 2009), low functioning autism (Chan, Sze, Cheung, Han, Leung, & Shi, 2011) and Asperger’s syndrome (Chan, Sze, & Shi, 2008) have also revealed clinically significant improvement in various cognitive functions, emotional / behavioral problems as well as overall quality of life after 3 to 8 months of DMBI. The improved cognitive functions include memory, executive functions, language ability and impulse control ability. Behaviorally, the patients became less impulsive with better temper control, and more flexible in problem solving ability.

*On normally developed individuals*. This includes a randomized controlled EEG experiment to examine patterns of brain activation during *Dan Tian* breathing, an important component of the DMBI (Chan, Cheung, Sze, Leung, & Shi, 2011). In this study, 44 normal adults were randomly assigned to receive training on the Shaolin *Dan Tian* breathing (DTB) or the progressive muscle relaxation (PMR) for one month. It was found that after one-month training, the DTB group showed significantly enhanced EEG alpha asymmetry (an index of relaxation and positive mood) and theta coherence (an index of attention and alertness) after performing the DTB, which was not observed in the PMR group. This finding is suggestive of the positive effect of Shaolin DTB technique on enhancing human neural activity and connectivity, which may possibly enhance mood state and cognitive functions.

**3. RESEARCH PLAN AND METHODOLOGY**

The present study will use a randomized controlled trial design, and using tools including objective neuropsychological assessments and electroencephalographic measures, and subjective parents’ report on the behavioral changes, to examine the effect of DMBI in comparison with conventional cognitive and/or behavioral treatment on improving cognitive, emotional and behavioral functions of patients with brain disorders, and the neurophysiological effects on the brain.

## Participants

About 80 individuals (aged 6 to 17 years) with neurological disorders which may include ASD, ADHD, epilepsy, or stroke that have cognitive and behavioral dysfunction will be recruited for this study. The final patient group will be determined according to accessibility. The subjects will be recruited from the existing subject database of the Neuropsychology Laboratory of the co-investigators, and referrals from schools and public or private clinics in Hong Kong. Patients with severe motor dysfunctions that prevent them from participating in the treatment/assessment, and those with history of comorbid neurological and psychiatric disorders and head trauma will be excluded from the study. The rationale and the potential risk of the experiment will be explained to all subjects and/or their parents, and consent form will be obtained from all the parents of subjects. Each subject can withdraw from the study at any time they preferred.

## Study Design

## A randomized controlled trial design will be used in this study. While half of the participants will be randomly assigned to receive training on DMBI, the other half of participants will be assigned to receive conventional cognitive and /or behavioral treatment. The choice of cognitive or behavioral intervention will depend on the level of general intelligence of the participants recruited. Cognitive-behavioral therapy would be selected for participants with normal intellectual functioning, whereas behavioral therapy would be selected for participants with mental retardation. Baseline data will be collected within five weeks prior to the intervention. Intervention will then commence twice per week, for 4 weeks (1 hour / session), after which post-assessment will be done.

## Intervention

## The DMBI and cognitive / behavioral therapy will be administered by two clinical psychologists, who have over 10 years of clinical experience. The two treatment groups will be conducted in parallel sessions. During the one-month intervention period, each participant and his/her parent(s) or caretaker will receive training on group basis for 8 sessions, 1 hour per session. Both intervention groups will also be encouraged to apply the treatment techniques into their daily life practice.

*Dejian Mind-Body Intervention.* Each participant will be encouraged to adopt a special vegetarian diet and to largely reduce / refrain from hot and spicy foods. It is because in the Shaolin medical approach, these hot and spicy foods are believed to generate excessive heat inside the body if not digested well, which will in turn adversely affect the mind and the body of an individual. In addition, the participants will learn some basic traditional Shaolin mind-body exercises, including Dan Tian breathing, shoulder relaxation, tranquil stand and nasal bridge massage. Regular practice of these mind-body exercises can facilitate blood and Qi circulation and strengthen the mind and the body. Finally, parents of participants will be guided individually to change their negative thoughts based upon the *Chan* principles – i.e., understanding that the roots of all problems were greed, anger and obsessive thinking, faulty realization of the self and nature. This aims at alleviating the psychological distress of participants and their parents.

*Cognitive-Behavioral / Behavioral Therapy.* Symptom-specific cognitive-behavioral or behavioral therapy will be adopted depending on the characteristics of the recruited participants. For participants with normal intellectual functioning, some cognitive treatment techniques (e.g., cognitive restructuring) that aim to changing maladaptive thinking will be adopted. Given the limited cognitive abilities of participants with mental retardation, the treatment for those participants will be more behaviorally based. Techniques of behavioral modification will be introduced to and practiced with the children and their caregivers, so as to increase positive behaviors and to reduce disruptive behaviors. Similar arrangement as that for DMBI in terms of group-basis therapy with the involvement of parent or caregiver of the participant will be planned.

## Measures

Each participant will be administered the neuropsychological assessment and EEG testing, and his/her parent will be interviewed and asked to rated on the child’s behaviors by the clinical psychologist or trained research assistant at the PolyU. While the child will be assessed for a total of 3 hours, the parent will be interviewed and perform the behavioral rating for a total of 1 hour.

*Neuropsychological assessment*. Participants will be administered a neuropsychological battery that assesses different aspects of cognitive functioning that may include attention, planning, organization, flexible thinking, impulse control ability and memory. The neuropsychological assessment will be administered in paper-and-pencil format and the questions will be verbally delivered. The entire battery will take about 90 minutes to administer, and is planned to include the following:

1. The Wechsler Intelligence Scale for Children-Forth Edition (Hong Kong) (WISC-IV(HK)). The abbreviated version of the WISC-IV(HK) (Wechsler, 2010), which composed of four subtests (Similarities, Digit Span, Coding and Matrix Reasoning), will be used in the study to estimate the level of general intelligence of the children. The estimated IQ score has a mean of 100 and a standard deviation of 15.
2. D2 Test of Concentration (D2; Brickenkamp, 1981**).** It is a test of attention and inhibitory control involving 14 lines of letter ‘d’ or ‘p’ with a different number of dashes above and/or below the letter. The individual is required to cancel as many ‘d’s with 2 dashes as possible within 20 seconds for each line, while ignoring the non-target distractors.
3. Hong Kong List Learning Test. The HKLLT (2^nd^ ed.; Chan, 2006) will be use to measure the frontal lobe functions of learning strategies, organization, and vulnerability to interference.
4. Go/No-Go Task. It is a computerized task for testing the ability to attending to and flexibly responding to changing stimuli and to inhibit unwanted responses. A total of 192 black balls and 48 red balls (black:red ratio = 4:1) are randomly displayed, one at a time, in the center of the computer screen. The individual is required to press a key as quickly as possible in response to a black ball (Go stimulus), but to inhibit their responses when a red ball (No-Go stimulus) appeared.
5. Children’s Color Trail Test (CCTT; Williams et al., 1995). It is a test of mental flexibility, which involves duplicates of each number embedded within pink and yellow circles, and requires to connect the numbers in ascending order from 1 to 15 while alternating between the two colors as quickly as possible.
6. The Five Point Test (FPT; Regard et al., 1982). This is another test for mental flexibility, which requires spontaneous generation of novel designs without repetition by connecting five points with straight lines within 5 minutes.
7. The Tower of California Test (ToC; Mattson et al., 1999**).** The ToC is a common test for planning ability and strategy formulation. It consists of nine items that involve moving dics on three colored vertical pegs to match a target arrangement while adhering to rules.

*EEG*. The EEG test and the neuropsychological tests will be carried out at the PolyU under the supervision of the Principal Investigator who is a clinical neuropsychologist. During the EEG test, each subject will be put on a 19-channel EEG cap, and their EEG signal recorded during resting state for 5 minutes and during the Go/No-Go test.

*Behavioral Measures.* Parents of each participant will serve as the informants during the structured interview based on standardized questionnaires on the developmental history of the participant and on their daily behavioral problems before and after the intervention. The parents of the intervention group will also be required to evaluate the degree of changes in behaviors after intervention.

# Procedure

The neuropsychological assessment, EEG recording and parents’ evaluation will be done before and after the intervention at the PolyU. The neuropsychological assessment will take about 90 minutes, where participants will be assessed individually in a quiet room by a clinical psychologist. The EEG recording will be done by an EEG technician and the recording which will take about 90 mins. The interview with parents will be conducted by a clinical psychologist or a trained research assistant at a separate room, which will take about 60 minutes. All participants and their parents will be informed of the nature of the study, their right to withdraw at any time during the study. Written informed consent will be obtained prior to study participation. The study will be conducted according to the Declaration of Helsinki.

After the baseline assessment, the DMBI group will be run by the first Co-Investigator (a clinical psychologist who has developed the DMBI), while the cognitive / behavioral therapy group will run by a clinical psychologist who has over 10 years of clinical experience in treating pediatric clinical population. The intervention will last for four weeks (twice per week), and each participant and his/her parent will be arranged for post-assessment.

# Data Analyses

*Neuropsychological assessment*. Participants’ performance on the neuropsychological battery will be scored according to standardized scoring procedures. ANOVA with repeated measures will be performed to compare the change in test scores between the two intervention groups (DMBI vs Cognitive / Behavioral therapy) after the one-month intervention.

*EEG data*. The EEG signals will be recorded onto a computer, digitized, filtered, edited for artifacts, and fourier transformed for data analysis. Group comparisons on the different indices will be conducted between participants in the two groups using ANOVA with repeated measures.

*Behavioral measures*. Participants’ parental rating on standardized behavioral measures will be scored according to standardized scoring procedures. ANOVA with repeated measures will be performed to compare the change in rating between the two intervention groups (DMBI vs Cognitive / Behavioral therapy) after the one-month intervention.

Schedule

The project is expected to be completed in 18 months, commencing in November 2012 and completed by May 2014.

| Time-line | Research Activities |
| --- | --- |
| 1 – 2 months | Literature review, hiring staff, recruitment of participants |
| 2 – 3 months | Preparation of EEG-recording equipment and paradigms, neuropsychological battery, training of staff |
| 4 – 5 months | Baseline data collection |
| 6 – 7 months | One-month Intervention |
| 8 – 9 months | Post-one-month-intervention assessment |
| 10 – 18 months | Data processing, data analysis, recruitment of possible new patient groups for study |

References

Benson, H., Rosner, B. A., Marzetta, B. R., & Klemchuk, H. M. (1974). Decreased blood-pressure in pharmacologically treated hypertensive patients who regularly elicited the relaxation response. *Lancet 23*, 1(7852), 289-291.

Brickenkamp, R. (1981). Aufmerksamkeits—Belastungs-test, test d2. Gottingen: Hogrefe.

Caudill, M., Schnable, R., Zuttermeister, P., Benson, H., & Friedman, R. (1991). Decreased clinic utilization by chronic pain patients after behavioral medicine intervention. *Pain, 45,* 334-335.

Chan AS. Hong Kong List Learning Test, 2^nd^ ed. Hong Kong: Department of Psychology and Clinical Psychology Centre, The Chinese University of Hong Kong, 2006.

Chan, A. S., Cheung, M. C., & Sze, S. L. (2007). Effect of mind/body training on children with behavioral and learning problems: A randomized controlled study. In B.N. DeLuca (Ed.), *Mind-body and relaxation research focus*. Hauppauge, NY: Nova Science.

Chan, A.S., Cheung, M.C., Sze, S.L., Leung, W.W. & Shi, D. (2011). Shaolin Dan Tian Breathing fosters relaxed and attentive mind: a randomized controlled neuroelectrophysiological study. Evidence-based Complementary and Alternative Medicine, DOI:10.1155/2011/180704.

Chan, A.S., Cheung, M.C., Tsui, W.J., Sze, S.L., & Shi, D. (2011). Dejian mind-body intervention on depressive mood of community-dwelling adults: a randomized controlled trial. *Evidence-based Complementary and Alternative Medicine,* DOI:10.1093/ecam/nep043.

Chan, A. S., Han, Y. M. Y., & Cheung, M. C. (2008). Electroencephalographic (EEG) measurements of mindfulness-based Triarchic Body-Pathway Relaxation Technique: A pilot study. *Applied Psychophysiology and Biofeedback, 33*, 39-47

Chan, A. S., Ho, Y. C., Cheung, M. C., Albert, M. S., Chiu, H. F. K., & Lam, L. C. (2005). Association between mind-body and cardiovascular exercises and memory in older adults. *Journal of the American Geriatrics Society, 53*, 1754-1760.

Chan, A.S., Sze, S.L., Cheung, M.C., Han, Y.M.Y., Leung, W.W.M. & Shi, D. (2011). Dejian mind-body intervention improves the cognitive functions of a child with autism. Evidence-based Complementary and Alternative Medicine, DOI:10.1155/2011/549254.

Chan, A.S., Sze, S.L., Cheung, M.C., Lam, J.M.K., & Shi, D. (2009). Dejian mind-body intervention improves the functioning of a patient with chronic epilepsy: a case report. Cases Journal, DOI:10.1186/1757-1626-2-9080.

Chan, A.S., Sze, S.L., Han, Y. M. Y., & Cheung, M. C. (In Press). A Chan dietary intervention enhances executive functions and anterior cingulate activity in autism spectrum disorders: a randomized controlled trial. *Evidence-based Complementary and Alternative Medicine.*

[Chan, A.S., Sze, S.L., & Shi, D. (2008). Traditional Chinese mind-body exercises improve self control ability of an adolescent with Asperger's disorder](http://www.cuhk-inrc.com/attachments/File/DMBI_asperger.pdf). [*Journal of Psychology in Chinese Societies, 9,* 225-23](http://www.cuhk-inrc.com/attachments/File/DMBI_asperger.pdf)9.

Chan, A.S., Wong, Q.Y., Sze, S.L., Kwong, P.P.K., Han, Y.M.Y., Cheung, M.C. (2012). A Chinese Chan-based mind-body intervention improves sleep on patients with depression: a randomized controlled trial. *The Scientific World Journal,* DOI: 10.1100/2012/235206.

Chan, A.S., Wong, Q.Y., Sze, S.L., Kwong, P.P.K., Han, Y.M.Y., Cheung, M.C. (In Press). A Chinese Chan-based mind-body intervention for patients with depression. *Journal of Affective Disorders*.

Chou, K. L., Lee, P. W. H., Yu, E. C. S., Macfarlane, D., Cheng, Y. H., Chan, S. S. C., & Chi, I. (2004). Effect of Tai Chi on depressive symptoms amongst Chinese older patients with depressive disorders: A randomized clinical trial. *International Journal of Geriatric*

Compton, S. N., March, J. S., Brent, D., Albano, A., M., Weersing, R., & Curry, J. (2004). Cognitive-behavioral psychotherapy for anxiety and depressive disordersin children and adolescents: an evidence-based medicine review. *Journal of the American Academy of Child & Adolescent Psychiatry. 43,* 930-959.

Deckro, G. R., Ballinger, K. M., Hoyt, M., Wilcher, M., Dusek, J., Myers, P., Greenberg, B., Rosenthal, D. S., & Benson, H. (2002). The evaluation of a mind/body intervention to reduce psychological distress and perceived stress in college students. *Journal of American College Health, 50*, 281-287.

Jacobs, G. D. (2001). Clinical applications of the relaxation response and mind/body interventions. *Journal of Alternative and Complementary Medicine.* *7,* S3-S101.

Jacobs, G. D., Benson, H., & Friedman, R. (1993). Home-based central nervous system assessment of a multifactor behavioral intervention for chronic sleep-onset insomnia. *Behavioral Therapy, 24,* 159-174.

Jin, P. (1989). Changes in heart rate, noradrenaline, cortisol and mood during Tai Chi. *Journal of Psychosomatic Research, 33(2),* 197-206.

Jin, P. (1992). Efficacy of Tai Chi, brisk walking, meditation, and reading in reducing mental and emotional stress. *Journal of Psychosomatic Research, 36(4),* 361-370.

Klein, P. J., & Adams, W. D. (2004). Comprehensive therapeutic benefits of Taiji: A critical review. *American Journal of Physical Medicine & Rehabilitation, 83(9),* 735-745.

Lai, J. S., Lan, C., Wong, M. K., & Teng, S. H. (1995). Two-year trends in cardiorespiratory function among older Tai Chi Chuan practitioners and sedentary subjects. *Journal of the American Geriatrics Society, 43(11),* 1222-1228.

Lan, C., Chen, S. Y., Lai, J. S., & Wong, M. K. (1999). The effect of Tai Chi on cardiorespiratory function in patients with coronary artery bypass surgery. *Medicine & Science in Sports & Exercise, 31(5),* 634-638.

Lang, R., Regester, A., Lauderdale, S., Ashbaugh, K., & Haring, A. (2010). Treatment of anxiety in autism spectrum disorders using cognitive behavior therapy: a systematic review. *Developmental Neurorehabilitation, 13,* 53-63.

Leserman, J. Stuart, E. M., Mamish, M. E., & Benson, H. (1989). The efficacy of the relaxation response in preparing for cardiac surgery. *Behavioral Medicine, 15,* 111-117.

Li, F., Duncan, T. E., & Duncan, S. C. (2001). Enhancing the psychological well-being of elderly individuals through Tai Chi exercise: A latent growth curve analysis. *Structural Equation Modeling, 8(1),* 53-83.

Li, F., McAuley, E., Harmer, P., Duncan, T. E., & Chaumeton, N. R. (2001). Tai Chi enhances self-efficacy and exercise behavior in older adults. *Journal of Aging and Physical Activity, 9,* 161-171.

Mandle, C. L., Jacobs, S. C., Arcari, P. M., & Domar, A. D. (1996). The efficacy of relaxation response interventions with adult patients: a review of the literature. *Journal of Cardiovascular Nursing, 10,* 4-26.

Mattson, S. N., Goodman, A. M., Caine, C., Delis, D. C., & Riley, E. P. (1999). Executive functioning in children with heavy prenatal alcohol exposure. *Alcoholism:Clinical and Experimental Research, 23,* 1808–1815.

Moree, B. N., & Davis III, T. E. (2010). Cognitive-behavioral therapy for anxiety in children diagnosed with autism spectrum disorders: modification trends. *Research in Autism Spectrum Disorders, 4,* 346-354.

Nakao, M., Fricchione, G., Myers, P., Zuttermeister, P. C., Barsky, A. J., & Benson, H. (2001). Depression and [education](http://searchmiracle.com/text/search.php?qq=Education) as predicting factors for completion of a behavioral medicine intervention in a mind/body medicine clinic. *Behavioral Medicine, 26*, 177-184.

Piacentini, J., Woods, D. W., Scahill, L., Wilhelm, S., Peterson, A. L., Chang, S., Ginsburg, G. S., Deckersbach, T., Dziura, J., Levi-Pearl, S., & Walkup, J. T. (2010). Behavior therapy for children with Tourette disorder: a randomized controlled trial. *The Journal of the American Medical Association, 303,* 1929-1937.

Regard, M., Strauss, E., & Knapp, P. (1982). Children’s production on verbal and non-verbal fluency tasks. *Perceptual and Motor Skills, 55,* 839–844.

Sandlund, E. S., & Norlander, T. (2000). The effects of Tai Chi Chuan relaxation and exercise on stress responses and well-being: An overview of research. *International Journal of Stress Management, 7(2),* 139-149.

Schwartz, J. M., & Begley, S. (2002). *The mind and the brain. Neuroplasticity and the power of mental force.* New York : Regan Books.

Tsang, H.W.H., Cheung, L., & Lak, D. (2002). Qigong as a psychosocial intervention for depressed elderly with chronic physical illnesses. *International Journal of Geriatric Psychiatry,* *17,* 1146-1154.

Wang, Y. T., Taylor, L., Pearl, M., & Chang, L. S. (2004). Effects of Tai Chi exercise on physical and mental health college students. *American Journal of Chinese Medicine, 32(3),* 453-459.

Waxmonsky, J. G., Waschbusch, D. A., Pelham, W. E., Draganac-Cardona, L., Rotella, B., & Ryan, L. (2010). Effects of atomoxetine with and without behavior therapy on the school and home functioning of children with attention-deficit/hyperactivity disorder. *Childhood and Adolescent Mental Health, 71,* 1535-1551.

Wechsler D. (2010). *Wechsler Intelligence Scale for Children – Fourth Edition (Hong Kong).* China: King-May Psychological Assessment Ltd.

Williams, J., Rickert, V., Hogan, J., Zolten, A. J., Satz, P., D’Elia, L. F., et al. (1995). Children’s color trails. *Archives of Clinical Neuropsychology, 10,* 211–223.

Yeh, G. Y., Wood, M. J., Lorell, B. H., Stevenson, L. W., Eisenberg, D. M., Wayne, P. M., Goldberger, A. L., Davis, R. B., & Phillips, R. S. (2004). Effects of Tai Chi mind-body movement therapy on functional status and exercise capacity in patients with chronic heart failure: A randomized controlled trial. *The American Journal of Medicine, 117*, 541-548.

Young, D. R., Lawrence, A., Jee, S. H., & Miller, E. R. III. (1999). The effects of aerobic exercise and Tai Chi on blood pressure in older people: Results of a randomized trial. *Journal of the American Geriatrics Society, 47(3),* 277-284.

**4. PROJECT FUNDING**

The project is currently funded by a private donation.

**5. PLANS FOR COLLABORATION**

Dr. Mei-chun Cheung of the Institute of Textiles and Clothing of The Hong Kong Polytechnic University will be responsible for participant recruitment, and supervising analysis on the neuropsychological and EEG data. She will also be involved in the development of theoretical background of the study and in designing the EEG experimental paradigms.

Prof. Agnes S. Chan of the Department of Psychology of The Chinese University of Hong Kong will be responsible for developing the theoretical background of the study, designing the experimental paradigm, and developing the protocol to be used. She will also supervise the trainers for providing the intervention throughout the study, the research assistants in analyzing the data collected, and conceptualize the analyses results for manuscript preparation.

Dr. Sophia L. Sze of the Department of Psychology of The Chinese University of Hong Kong will be responsible for designing, coordinating and running the experiment, training and supervising the research assistant in data collection and management. She will also be responsible for data analysis and manuscript preparation.

Dr. Yvonne M. Han of the Department of Special Education and Counselling of The Hong Kong Institute of Education will be responsible for supervising the data collection process and data analysis.

**6. ETHICAL CONSIDERATIONS**

The research will be conducted according to the requirements stated in the Helsinki Declaration. All participants will participate voluntarily, and can withdraw from the study at any time. All personal information and samples collected will be kept strictly confidential, and the protocol and data will be kept in a locked cabinet accessible only to the research personnel involved.
